# Impact of the COVID-19 outbreak on severe trauma trends and healthcare system reassessment in Lombardia, Italy: an analysis from the regional trauma registry

**DOI:** 10.1186/s13017-021-00383-y

**Published:** 2021-07-19

**Authors:** Riccardo Giudici, Armando Lancioni, Hedwige Gay, Gabriele Bassi, Osvaldo Chiara, Claudio Mare, Nicola Latronico, Antonio Pesenti, Roberto Faccincani, Luca Cabrini, Roberto Fumagalli, Arturo Chieregato, Laura Briani, Fabrizio Sammartano, Giuseppe Sechi, Alberto Zoli, Andrea Pagliosa, Giuseppe Foti, Erika Borotto, Alessandra Palo, Oliviero Valoti, Marco Botteri, Michele Carlucci, Elisa Reitano, Roberto Bini

**Affiliations:** 1 Department of Anesthesia and Intensive Care Medicine, ASST Niguarda, Milan, Italy; 2 Department of Anesthesia and Intensive Care Medicine, University Milano Bicocca, Monza, Italy; 3 Emergency Department, General Surgery and Trauma Team, ASST Niguarda, University of Milano, Piazza Ospedale Maggiore 3, 20162 Milano, Italy; 4 Regional Agency of Emergency and Urgency, Milan, Italy; 5 Department of Medical and Surgical Specialties, Radiological Sciences and Public Health, University of Brescia, Brescia, Italy; 6 Department of Anesthesia, Critical Care and Emergency, Spedali Civili University Hospital, Brescia, Italy; 7 Department of Anesthesia, Critical Care and Emergency, Fondazione Policlinico, University of Milan, Milan, Italy; 8 Emergency Department, Ospedale San Raffaele, Milan, Italy; 9 Department of Anesthesia and Intensive Care, Ospedale di Circolo e Fondazione Macchi, University of Insubria, Varese, Italy; 10 Department of Anesthesia and Intensive Care Medicine, University Milano Bicocca, Milan, Italy; 11 Department of Anesthesia and Intensive Care Medicine, Neuro Intensive Care, ASST Niguarda, Milan, Italy; 12 Emergency Department, Department of General Surgery and Trauma Team, ASST Niguarda, Milan, Italy; 13 Emergency Department, Emergency and Trauma Surgery, ASST Santi Carlo e Paolo, Milan, Italy; 14 Department of Anesthesia and Intensive Care Medicine, S.Gerardo Hospital, University Milano Bicocca, Monza, Italy; 15 Department of Anesthesia and Intensive Care, Ospedale di Circolo e Fondazione Macchi, Varese, Italy; 16 Regional Agency of Emergency and Urgency, Pavia, Italy; 17 Regional Agency of Emergency and Urgency, Bergamo, Italy; 18 Regional Agency of Emergency and Urgency, Brescia, Italy; 19 General and Emergency Surgery and Emergency Department, Ospedale San Raffaele, Milan, Italy; 20 Department of Translational Medicine, University of Eastern Piedmont, Novara, Italy

**Keywords:** Trauma, Emergency, COVID-19, Trauma care

## Abstract

**Backgrounds:**

The COVID-19 pandemic drastically strained the health systems worldwide, obligating the reassessment of how healthcare is delivered. In Lombardia, Italy, a Regional Emergency Committee (REC) was established and the regional health system reorganized, with only three hospitals designated as hubs for trauma care. The aim of this study was to evaluate the effects of this reorganization of regional care, comparing the distribution of patients before and during the COVID-19 outbreak and to describe changes in the epidemiology of severe trauma among the two periods.

**Methods:**

A cohort study was conducted using retrospectively collected data from the Regional Trauma Registry of Lombardia (LTR). We compared the data of trauma patients admitted to three hub hospitals before the COVID-19 outbreak (September 1 to November 19, 2019) with those recorded during the pandemic (February 21 to May 10, 2020) in the same hospitals. Demographic data, level of pre-hospital care (Advanced Life Support-ALS, Basic Life Support-BLS), type of transportation, mechanism of injury (MOI), abbreviated injury score (AIS, 1998 version), injury severity score (ISS), revised trauma score (RTS), and ICU admission and survival outcome of all the patients admitted to the three trauma centers designed as hubs, were reviewed. Screening for COVID-19 was performed with nasopharyngeal swabs, chest ultrasound, and/or computed tomography.

**Results:**

During the COVID-19 pandemic, trauma patients admitted to the hubs increased (46.4% vs 28.3%, *p* < 0.001) with an increase in pre-hospital time (71.8 vs 61.3 min, *p* < 0.01), while observed in hospital mortality was unaffected. TRISS, ISS, AIS, and ICU admission were similar in both periods. During the COVID-19 outbreak, we observed substantial changes in MOI of severe trauma patients admitted to three hubs, with increases of unintentional (31.9% vs 18.5%, *p* < 0.05) and intentional falls (8.4% vs 1.2%, *p* < 0.05), whereas the pandemic restrictions reduced road- related injuries (35.6% vs 60%, *p* < 0.05). Deaths on scene were significantly increased (17.7% vs 6.8%, *p* < 0.001).

**Conclusions:**

The COVID-19 outbreak affected the epidemiology of severe trauma patients. An increase in trauma patient admissions to a few designated facilities with high level of care obtained satisfactory results, while COVID-19 patients overwhelmed resources of most other hospitals.

## Background

The COVID-19 epidemic started in Italy on February 21, 2020, with the first Italian case diagnosed in Codogno, a little town in Lombardia, a region of the Northern Italy. Lombardia has an area of 24,000 km^2^ (9302 square miles), with 9,737,074 residents (1046 persons per square mile) and at least one million daily commuters. As the spread of COVID-19 was rapid and devastating in this highly populated area, all emergency departments (ED) were suddenly put under pressure by an overwhelming number of patients with acute respiratory distress requiring intensive care unit (ICU) support. Lombardia was one of the first places in the world to be affected this drastically; thus, guidance for care of COVID-19 was largely non-existent. A Regional Emergency Committee (REC) was established and the regional health system reorganized. This reorganization included the interruption of elective surgical activities in order to increase the number of available ward beds and redirect the time of the anesthesiology staff, who began converting operating and recovery rooms into additional intensive care unit beds [1]. A new distribution of patients with time-dependent pathologies, such as trauma, ST-elevation myocardial infarction (STEMI), and stroke, were then designated to a few hospitals considered as hubs. In the “pre-COVID-19” era, the regional trauma network had six level 1 and 18 level 2 trauma centers, all with neurosurgical services. Based on previous epidemiologic studies [2], 3800 major trauma per year are to be expected, 37% road-related and 75% requiring ICU admission. Due to the epidemic outbreak, the REC established a lockdown policy starting on March 8, and a decrease of road-related trauma patients was anticipated. The American College of Surgeons Committee on Trauma (ACS-COT) [3] guidelines for COVID response provided a general framework for the pandemic response, stating to preserve the hospital capacity for severe trauma patients through a reorganization of the regional systems.

In Italy, the trauma network was reorganized in order to funnel COVID-19 patients to defined locations with dedicated ICU resources and reallocate the regional trauma flow to ensure optimal trauma care.

Three adult level 1 trauma centers with all specialties available 24/7 and dedicated trauma care, and one referral center for pediatric trauma, were established as hubs for the major trauma of the region.

One adult center was in the mid-west, the second in the northwest and the third in the northeast (respectively, the cities of Milano, Varese, and Brescia). The pediatric center was in the middle of the region (Bergamo). Most level 2 trauma centers were partially or totally converted into COVID-19 hospitals. Pre-hospital triage rules of the Emergency Medical System (EMS) were established as follows: patients with unstable vital signs or evidence of a critical injury on scene were directly admitted to the closest level 1 trauma center, with an increased use of air ambulance; patients with normal vital signs who sustained a high energy mechanism of trauma and no evidence of critical injury were preferentially sent to the closest available level 2 trauma center.

Using the regional trauma registry, we analyzed data of trauma patients admitted to the three trauma centers designed as hubs during the pandemic, comparing the results with those of the same centers during the pre-COVID-19 period. The aim of the study was to evaluate how the trauma system reassessment was able to guarantee trauma access and care. Moreover, we evaluated how the COVID-19 outbreak modified the epidemiology of major trauma in our region.

## Methods

The Lombardia Trauma Registry (LTR) started in 2018 with a progressive addition of the emergency departments (ED) and dispatch centers of the region. This process was concluded in August 2019. The institution of LTR was approved by Ethics Committee Milano Area 2 on July 17, 2018 (record number 569/2018). Inclusion criteria of trauma patients in the LTR were (a) admission to a regional level 1 or 2 trauma center and (b) transport by EMS with pre-alert of hospital trauma team or (c) the presence at hospital evaluation of a critical injury.

Demographic data, level of pre-hospital care (advanced life support-ALS, basic life support-BLS), type of transportation, pre-hospital time, mechanism of injury (MOI), abbreviated injury score (AIS, 1998 version), injury severity score (ISS), revised trauma score (RTS), ICU admission and survival outcome of all the patients admitted to the trauma centers of Milano Niguarda, Varese Ospedale di Circolo, and Brescia Spedali Civili were retrospectively reviewed. Conventionally, an ISS < 16 was defined as minor trauma. Expected mortality was obtained by Trauma and Injury Severity Score (TRISS) system.

Motor vehicle collisions, motorcycle collisions, bicycle collisions, and pedestrians hit by a vehicle were defined collectively as road-related trauma. Moreover, according to the trauma intentionality, injuries were stratified as *intentional* (either self-inflicted or resulting from interpersonal violence) or *un-intentional* (road traffic, work related injuries, accidental falls and other accidental injuries) [4]. MOI was defined as “not classified” if the retrieval of MOI from the registry was not available. Patients pronounced dead on the scene and the MOI which caused the death were recorded whenever possible. Patients who were dead on arrival in the emergency department were considered in-hospital deaths.

Trauma patients were defined as “triage code 1” if unstable vital signs unresponsive to initial resuscitation were recorded on the scene, and “triage code 2” if unstable vital signs responsive to initial resuscitation or anatomy of critical injury were observed. “Triage code 3” patients had a high energy trauma mechanism without altered vital signs and no evidence of a major injury. Guidelines by the REC were to send codes 1 and 2 patients, whenever possible, to one of the three hubs and code 3 patients to the closest level 2 trauma center.

Data were analyzed with statistical software (R-cran). The sample distribution was evaluated with Kolmogorov-Smirnov and Shapiro-Wilk tests resulting in a non-Gaussian distribution for any of the examined variable. Continuous variables were compared using Mann-Whitney tests, while categorical variables were analyzed using Pearson’s Chi-squared test. A *p* value ≤ 0.05 was considered significant.

Two different periods of the same length of time (12 weeks) before and during the epidemic outbreak, with the participation to LTR of all regional areas, were compared. The period from February 21 to May 10, 2020, was indicated as the COVID-19 period, while the period from September 1 to November 19, 2019, and was defined as the no-COVID-19. The no-COVID-19 period was chosen because of the availability of data from all the region, since LTR activation was concluded on August 2019.

## Results

Though the absolute number of trauma patients transported by EMS was reduced by 35% during the COVID-19 outbreak, the percent of trauma patients admitted to the three hubs was significantly increased (Table 1).

**Table 1:** Demographic data of patients transported to the three hubs

	COVID-19	no COVID-19	P value
Total trauma transported by EMS	744	1150	
Total transported to the hubs [*n* (%)]	**345 (46.4)**	**325 (28.3)**	0.001*
Male [*n* (%)]	262 (75.9)	248 (76.3)	0.92
Age [median (IQR)]	48 (34-64)	43.0 (27-62)	0.017*
Penetrating [*n* (%)]	32 (9.3)	17 (5.2)	0.063
Triage code 1 [*n* (%)]	103 (29.9)	111 (34.2)	0.267
Triage code 2 [*n* (%)]	235 (68.1)	201 (61.8)	0.105
Triage code 3[*n* (%)]	7 (2.0)	13 (4.0)	0.204
Admitted [*n* (%)]	266 (77.1)	237 (72.9)	0.32
ISS (of admitted pts) [median (IQR)]	13 8-21.75	14 8-25	0.44
ISS < 16 [*n* (%)]	150 (56.4)	131 (55.3)	0.871
ISS 16-24 [*n* (%)]	61 (22.9)	46 (19.4)	0.393
ISS > 24 [*n* (%)]	55 (20.7)	60 (25.3)	0.258
Helicopter ALS with doctor [*n* (%)]	100 (29)	71 (21.8)	0.042*
Ground ALS with nurse [*n* (%)]	20 (5.8)	25 (7.7)	0.409
Ground ALS with doctor [*n* (%)]	136 (39.4)	155 (47.7)	0.037*
Ground BLS with paramedic [*n* (%)]	80 (23.2)	64 (19.7)	0.314
Transfer from other Hospital [*n* (%)]	9 (2.6)	10 (3.1)	0.895
AIS ≥ 3 head and neck [*n* (%)]	99 (37.2)	92 (34.6)	0.782
AIS ≥ 3 chest [*n* (%)]	103 (38.7)	88 (33.1)	0.783
AIS ≥ 3 abdomen [*n* (%)]	62 (23.3)	48 (18)	0.472
AIS ≥ 3 skeletal [*n* (%)]	106 (39.8)	95 (35.7)	1.000
ICU admission [*n* (%)]	92 (34.6)	98 (41.4)	0.142
Surgery < 24 h (%)	35 (13.2)	33 (13.9)	0.904
Died on ED [*n* (%)]	5 (1.9)	1 (0.4)	0.275
Died in the hospital [*n* (%)]	18 (6.8)	19 (8)	0.715
TRISS expected hospital mortality (%)	9.9	11.5	0.901
Observed hospital mortality (%)	6.8	8	0.634
Delta between observed and expected hospital mortality	−3.1	−3.5	1.000

*: ≤ 0.05

*EMS* emergency medical system, *ISS* injury severity score, *AIS* abbreviated injury scale, *ED* emergency department, *ALS* advanced life support, *BLS* basic life support, *TRISS* trauma and injury score

Subsequent data of Tables 1 and 2 concern exclusively the patients transported to the three adult trauma centers of the region.

**Table 2:** Mechanism of injury (MOI) and patients’ age during COVID-19 and no COVID-19 periods

	Age [median (IQR)]	No classified [*n* (%)]	Stab [*n* (%)]	Road related [*n* (%)]	Falls [*n* (%)]
	**COVID-19 period** (**345 patients**)				
Unintentional	50 (34-64)	28 (8.1)	6 (1.7)	123 (35.6)**	110 (31.9)*
Intentional (self inflicted)	36 (24.5-63)	1 (0.3)	10 (2.9)	-	29 (8.4)*
Intentional (by others)	44 (31.8-51)	1 (0.3)	19 (5.5)	-	-
Unknown	52 (40.5-76)	4 (1.1)	1 (0.3)	2 (0.6)	11 (3.2)
Total		34 (9.8)	36 (10.4)	125 (36.2)	150 (43.5)
	**no-COVID-19 period (325 patients)**				
Unintentional	43 (26.5-61.5)	21 (6.4)	3 (0.8)	195 (60)	60 (18.5)
Intentional (self inflicted)	37.5 (23.8-58.5)	3 (0.9)	5 (1.5)	-	4 (1.2)
Intentional (by others)	30 (24.4-31)	5 (1.5)	8 (2.5)	-	2 (0.6)
Unknown	63 (47.5-84)	2 (0.6)	1 (0.3)	1 (0.3)	15 (4.6)
Total		31 (9.5)	17 (5.2)	196 (60.3)	81 (24.9)

*p < 0.05; **p < 0.001

The median age was significantly higher during COVID-19. No difference in triage categories, median ISS, and percentage of ISS clusters was observed. Patients with minor trauma directly discharged from the ED or admitted with an ISS < 16, were 67% in the no COVID-19 period and 66% in the COVID-19. In the COVID-19 period, the number of patients transported with air ambulance was significantly higher with a decrease in the use of ground ambulances with doctors.

Data of the severity of patients admitted to the trauma centers and hospital outcome are summarized in the lower part of Table 1. No difference was observed between head, chest, abdomen, and skeletal injuries. The rate of ICU admissions and surgery within 24 h was similar and no difference was observed in hospital mortality, both in the ED and after admission. The observed hospital mortality was lower than the expected one calculated with TRISS, with a similar delta. Therefore, logistic regression model was not performed, given the few differences among the groups at the univariate analysis.

**Fig. 1:**
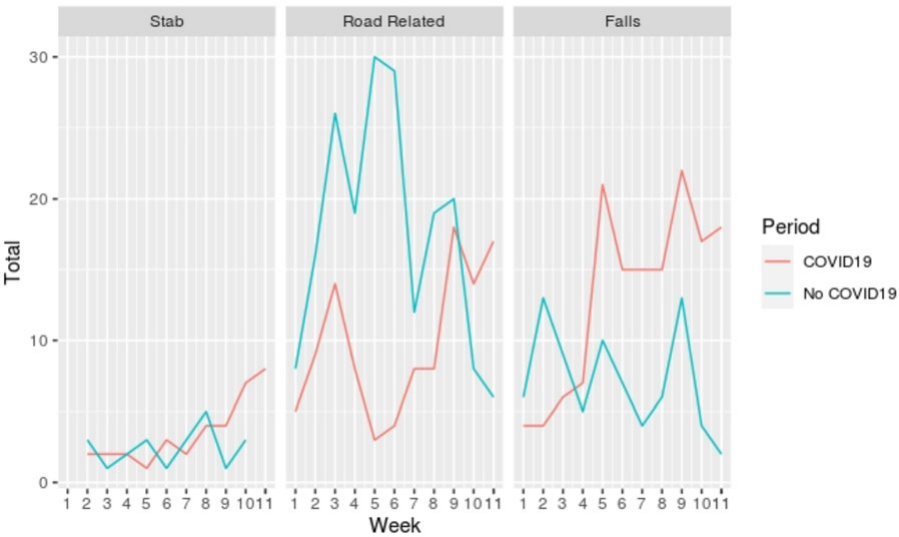
Trends of different mechanisms of trauma in the two compared periods

In the COVID-19 period, a consistent change of MOIs of severe trauma patients admitted to the three hubs was recorded (Table 2). During the lockdown, road-related trauma decreased, while an unexpected increase in falls was observed. Most falls occurred in the domestic setting and the number of intentional falls for suicide attempt increased sevenfold, compared with the no COVID-19 period. Stab wounds increased in the COVID-19 period, both self-inflicted and due to interpersonal violence, though not reaching statistical significance given the small number of cases. Differences of trends of MOIs during the 12 weeks of the two periods are shown in Fig. 1.

This difference of MOI is further strengthened by the analysis of pre-hospital deaths (Table 3).

**Table 3:** Deaths on the scene and total number of patients with unintentional and intentional injuries

Number of deaths	COVID-19 N° 744	no COVID-19 N° 1150	***P*** value
Total deaths on scene [*n* (%)]	130 (17.5)	78 (6.8)	< 0.001^a^
Unintentional road-related [*n* (%)]	21 (2.8)	20 (1.7)	0.138
Unintentional falls [*n* (%)]	12 (1.6)	6 (0.5)	0.899
Others unintentional [*n* (%)]	5 (0.7)	5 (0.4)	0.616
Intentional falls [*n* (%)]	14 (1.9)	6 (0.5)	0.627
Self inflicted stabs [*n* (%)]	10 (1.3)	5 (0.4)	0.945
Others self-inflicted [*n* (%)]	34 (4.6)	16 (1.4)	0.451
Stab from interpersonal violence [*n* (%)]	2 (0.2)	-	
Others from interpersonal violence [*n* (%)]	2 (0.2)	-	
Human intent unknown [*n* (%)]	30 (4.0)	20 (1.7)	0.802
Deaths on scene plus in hospital deaths for unintentional injuries [*n* (%)]	305 (41.0)	310 (26.9)	< 0.001^a^
Death on scene plus pts admitted to hospitals for intentional injuries [*n* (%)]	122 (16.4)	54 (4.7)	< 0.001^a^

^a^Statistical significance

Patients declared dead on scene were double during the COVID-19 period, with an increase of intentional injuries, both self-inflicted and from interpersonal violence. Pre-hospital mortality was 17.5% of all trauma patients assisted by EMS in COVID-19 period and only 6.8% in the no COVID-19 period.

When considering the sum of pre-hospital deaths with patients admitted to the hubs for intentional and unintentional injuries, a statistically significant increase in intentional injuries during the COVID-19 compared with the no-COVID-19 period (16% vs 4.7% of the trauma population) was observed.

Table 4 describes pre-hospital time, from first call to arrival of basic life support (BLS) crew on scene, from BLS arrival to departure from scene, transportation time, from first call to advanced life support (ALS) crew arrival on scene, from BLS to ALS arrival on scene, total pre-hospital time (with and without the time of BLS arrival). Pre-hospital time was significantly longer in the COVID-19 period, for both higher time on scene and transportation time.

**Table 4:** Out of hospital time in minutes

	COVID-19			No COVID-19		
	IQ 25	Median	IQ 75	IQ 25	Median	IQ 75
Call-BLS	9.0	12.0	16	7	10.0	12.0
BLS on scene	26.0	37.0	49	23	32.0	44.0
Transport	10.0	14.0	22	7	11.0	18.0
Call-ALS	15.0	19.0	27	11	15.0	21.0
BLS-ALS	2.0	5.0	12	2	4.5	9.2
PH time (total)	55.2	68.5	83	48	58.0*	70.0
PH time (without BLS time)	43.0	54.0	70	37	46.0*	60.0

*PH* pre-hospital, *ALS* advanced life support, *BLS* basic life support. *p ≤ 0.01

## Discussion

Due to the COVID-19 epidemic outbreak, most of the medical resources were shifted away from the standard activities and allocated to the care of COVID-19 patients. A big challenge of the regional health systems was to maintain an adequate level of care facilities open for non-deferrable pathologies, such as strokes, STEMI, and trauma. The present study describes the adaptive strategy of the Lombardia trauma system and changes in the epidemiology of severe trauma patients during the epidemic. This study is the first one using the regional-based registry in the area most affected by the epidemic in Italy.

This study showed how the reassessment of regional trauma system during the COVID-19 pandemic through the centralization of trauma patients to three specialized hubs ensured high level of care despite the longer transport times. The number of trauma patients transported to the three hubs and the number of admissions after ED evaluation increased compared with the no-COVID period, without significant changes in the severity of injuries and hospital mortality. In the three hubs, the staff of doctors and nurses was increased with the temporary transfer of health care personnel from non-hub facilities.

During the COVID-19 outbreak, 95% of patients transported to the three hubs were in triage code 1 or 2, with an increased use of helicopter transportation. The triage categories derived from the Trauma System of Northern French Alps (TRENAU) [5] are recommended by the Italian Ministry of Health because of the high sensitivity and good specificity [6]. The aliquot of admitted patients with ISS < 16 was still high and this observation suggests the need of further training of our emergency trauma system with the new triage rules to decrease over triage. It is important to mention that ISS alone is an anatomical index, while the severity of patient should be evaluated also with other indicators, such as need of direct transfer of the patient from ED to OR or ICU.

This study showed epidemiologic changes in trauma mechanisms of patients admitted to the three hubs during the pandemic, demonstrating an increase of intentional injuries, in particular falls for suicide attempt, with a decrease of road-related trauma. It is important to underline that our data exclusively look at the three level 1 trauma centers active during the pandemic, while there is no information on the patients admitted to level 2 hospitals. However, a major trauma patient without EMS involvement and admitted to a non-hub hospital was unlikely because all ICU beds in these facilities were used for COVID patients.

The decrease of injuries due to road-related causes and incidents at the workplace was expected due to the lockdown. Normally these two mechanisms account for 37% of major trauma in Lombardia [2], and this was roughly the rate of decrease in trauma patients assisted by EMS. An Italian surgical survey showed that trauma care has decreased almost everywhere, but the number of trauma admissions was not reported [7]. Our results are consistent with reports of other countries. Hernigou et al. reported a reduction of 32% in orthopedic trauma admission in Belgium [8]. In a retrospective analysis of trauma admissions in Portsmouth New Hampshire (USA), a decrease in overall trauma admissions of 57.4% was noted, with an 80.5% decrease in motor-vehicle collisions [9]. In a report from a Chinese town [10], the lockdown reduced the volume of trauma patients by 39%, with a higher decrease in the number of male patients. Trauma patients returned to normal levels only after the end of pandemic peak and lockdown.

The change in epidemiology with an increase of intentional trauma confirms previous reports after natural disasters, defined as events in which a multitude of persons are contemporaneously exposed to a life-threatening situation. An increased interpersonal violence has been described after earthquakes, tsunamis, and hurricanes. The breakdown of the social security system and the increased violence of intimate partners against women have been considered to be possible causes [11]. Our data showed a trend toward an increase in penetrating trauma, principally stab wounds that occurred in the domestic setting. The most interesting observation of the present study was the marked increase of suicide attempts admitted to the hubs. This increase was mostly due to voluntary falls from height, as confirmed by data of pre-hospital deaths combined with those of hospital admissions. It has been described [12] that loss of family members or loved ones, personal threats of life, forced restriction of freedom, collapse of social cohesion, and the burden of economic derangement may cause an increase in depressive and anxious behaviors in at risk persons. In a study from Cleveland, Ohio (USA) [13], a higher prevalence of psychiatric disease (from 26 to 43%) among orthopedic trauma was observed during the peak of pandemic in the Winter and Spring 2020. It has been concluded that stress induced by COVID-19 produces a higher risk of dangerous behaviors in persons with mental illness. In general, these observations outline the need of preventive measures on at risk populations or persons exposed to emotive stress, including healthcare workers, as supported by Chinese and other Asian studies during the early phase of the pandemic [14, 15] and by European reports [16].

Our results are in-line with the study of Qasim Z. et al [17], who describes an effective reorganization of the trauma system during the COVID-19 period, showing also a decrease of road-related trauma and an increase in trauma due to self-inflicted or interpersonal violence.

The increase in falls during the COVID-19 period could explain in part the higher pre-hospital mortality. The number of intentional jumpers pronounced dead on scene during the COVID-19 period was more than twofold compared to the no-COVID-19 time, even if a significant difference was not observed due to the small number of observations. As already reported by other studies [18, 19], after a vertical deceleration trauma, intentional jumpers demonstrated a significantly higher mortality compared to accidental fallers (20.4% vs 5.2%).

Finally, the pre-hospital time was more prolonged during the COVID-19 period, with an increased use of air transportation because of the longer distance for centralization and ground ALS staff less available as they were engaged in intensive care services for the COVID-19 patients. Notwithstanding the increased pre-hospital time, the hospital mortality was unchanged. These data could reflect the effect of the high levels of care given by pre-hospital crews, mainly represented by doctors and nurses, and by specialized trauma centers. Indeed, observed survival was higher than the expected one with TRISS calculation and this result suggests a survival advantage in patients treated in the three trauma centers of the study. This advantage was maintained during the COVID-19 period to confirm a good performance of the regional trauma system notwithstanding the spread of pandemic. The reorganization of the regional trauma system was able to avoid an impairment of the delivery of trauma care due to the diversion of critical resources [20]. Therefore, the reorganization of the trauma systems in Lombardia during the COVID-19 period showed to be effective, showing no differences in outcome measures between the two periods. Indeed, our experience highlights the importance of remodeling access to trauma centers during a pandemic, to ensure the correct management of trauma emergency in specialized centers.

However, during the COVID-19 period, a general reduction of the absolute number of trauma patients transported to the three hubs was shown, while the severity was the same. Moreover, the healthcare staff of hubs was reinforced, with additional doctors and nurses. All these conditions probably contributed to maintain a high level of care in these hospitals.

This study has several limitations. First, it is a retrospective analysis from a regional trauma registry recently established, with all possible inaccuracies related to incorrect compilation of items.

Second, the study concerns only patients transported to the three level 1 trauma centers, while there is no information on those who were admitted to other hospitals of the trauma network. However, our results are mainly based on simple parameters easy to obtain or automatically recorded by EMS, such as outcome, place of death, type of transportation, triage code, and mechanism of injury, where an error is unlikely. Moreover, the primary criterion for including the patients in the regional trauma registry is the alert of EMS for a traumatic event and it is unlikely that in case of a serious accident such an alert is not implemented or that EMS would transport the patient to a non-hub hospital.

Finally, another limitation of this study is that we compared two different seasons, with possible bias in MOI distribution. The regional trauma registry started for all the region only in August 2019; therefore, previous data were not available. Nevertheless, the epidemiology of major trauma in the Lombardia region is not different in Autumn compared with Spring, as shown in previous studies [2].

## Conclusions

In conclusion, our results show that reassessment measures are needed to maintain trauma center access and trauma team safety while also caring for other critically ill patients. Therefore, this important lesson should be taken into account during future emergency periods such as this one.

The emergency lockdown during the COVID-19 pandemic in Lombardia led to a reduction of major trauma, especially road-related injuries. The number of patients with intentional injuries admitted to the active level 1 trauma centers was greatly increased during the lockdown and this result would merit further analysis to assess the role of pre-existing factors and their interaction with the imposed restrictions. An increase in centralization to fewer facilities with high level of care obtained satisfactory results in the capability of the health system to take care of trauma emergencies while COVID-19 patients overwhelmed resources of most hospitals.

## Data Availability

The datasets used and/or analyzed during the current study are available from the corresponding author on reasonable request.

## Abbreviations

ACS-COT: American College of Surgeons Committee on Trauma
AIS: Abbreviated injury score
ALS: Advanced life support
BLS: Basic life support
ED: Emergency department
EMS: Emergency medical system
ICU: Intensive care unit
ISS: Injury severity score
LTR: Regional Trauma Registry of Lombardia
MOI: Mechanism of trauma
OR: Operating room
REC: Regional Emergency Committee
RTS: Revised trauma score
TRISS: Trauma and injury severity score

## References

[1] Grasselli G, Pesenti A, Cecconi M (2020). Critical care utilization for the COVID-19 outbreak in Lombardy, Italy. Early experience and forecast during an emergency response. JAMA.

[2] Chiara O, Mazzali C, Lelli S, Mariani A, Cimbanassi S (2013). A population based study of hospitalized seriously injured in a region of northern Italy. World J Emerg Surg..

[3] O’Neill P, Prince J, Simon R, Teperman S, Winchell R, Bank M (2020). Early report from the greater New York chapter of the American College of Surgeons Committee on Trauma on the COVID-19 crisis.

[4] Ringdal KG, Coats TJ, Lefering R, Di Bartolomeo S, Steen PA, Røise O (2008). The Utstein template for uniform reporting of data following major trauma: a joint revision by SCANTEM, TARN, DGU-TR and RITG. Scand J Trauma Resusc Emerg Med.

[5] Bouzat P, Ageron FX, Brun J, Levrat A, Berthet M, Rancurel E (2015). A regional system to optimize the pre-hospital triage of trauma patients. Critical Care..

[6] Italian Ministry of Health, National Center for clinical excellence (CNEC). Guidelines for the integrated management of major trauma, from the scene to definitive care. https://snlg.iss.it/wp-content/uploads/2020/06/LGTM_Racc1_4_def.pdf.

[7] Cozza V, Fransvea P, La Greca A, De Paolis P, Marini P, Zago M (2020). I-ACTSS-COVID-19-the Italian acute care and trauma surgery survey for COVID-19 pandemic outbreak. Updates in Surgery..

[8] Hernigou J, Morel X, Callewier A, Bath O, Hernigou P (2020). Staying home during COVID 19 decreased fractures, but trauma did not quarantine in one hundred and twelve adults and twenty eight children and the tsunami of recommendations could not lockdown twelve elective operations. Int Orthop..

[9] Kamine TH, Rembisz A, Barron RJ, Kromer M (2020). Decrease in trauma admissions with COVID-19 pandemic. Western J Emerg Med..

[10] Yang F, Lu X (2020). The effect of COVID-19 on trauma system in one city of China. Scand J Trauma Resusc Emerg Med.

[11] Anastario M, Shehab N, Lawry L (2009). Increased gender-based violence among women internally displaced in Mississipi 2 years post-Hurricane Katrina. Disaster Med Public Health Prep..

[12] Rezaeian M (2008). Epidemiology of suicide after natural disasters: a review on the literature and a methodological framework for future studies. Am J Disaster Med..

[13] Ohliger E, Umpierrez E, Buehler L, Ohliger AW, Magister S, Vallier H, Hirschfeld AG (2020). Mental health of orthopaedic trauma patients during the 2020 COVID-19 pandemic. Int Orthop.

[14] Yao H, Chen JH, Xu YF. Patients with mental health disorders in the COVID-19 epidemic. Lancet Psychiatric. 2020;7(4):e21. 10.1016/S2215-0366(20)30090-0. 10.1016/S2215-0366(20)30090-0 PMC7269717 32199510

[15] Rajkumar RP (2020). COVID-19 and mental health: a review of existing literature. Asian J Psychiatr..

[16] Chevance A, Gourion D, Hoertel N, Llorca PM, Thomas P, Bocher R, et al. Ensuring mental health care during the SARS-CoV-2 epidemic in France: a narrative review. Encephale. 2020;46(3):193–201. 10.1016/j.encep.2020.03.001. 10.1016/j.encep.2020.04.005 PMC7174154 32370982

[17] Qasim Z, Sjoholm LO, Volgraft J, Sailes S, Nance ML, Perks DH (2020). Trauma center activity and surge response during the early phase of the COVID-19 pandemic—the Philadelphia story. J Trauma Acute Care Surg..

[18] Lapostolle F, Gere C, Borron SW, Pétrovic T, Dallemagne F, Beruben A, Lapandry C, Adnet F (2005). Prognostic factors in victims of falls from height. Crit Care Med..

[19] Casati A, Granieri S, Cimbanassi S, Reitano E, Chiara O. Falls from height. Analysis of predictors of death in a single center retrospective study. J Clin Med. 2020;9:3175. 10.3390/jcm9103175 PMC7601239 33007955

[20] Haut ER, Leeds IL, Livingston DH. The effect on trauma care secondary to the COVID-19 pandemic. Collateral damage from diversion of resources. Ann Surg. 2020; 272(3):e204-7. 10.1097/SLA.0000000000004105. 10.1097/SLA.0000000000004105 PMC7467027 32452950

